# Laparoscopic - assisted transpyelic rigid nephroscopy - simple alternative when flexible ureteroscopy is not available

**DOI:** 10.1590/S1677-5538.IBJU.2014.0588

**Published:** 2016

**Authors:** Marcos Tobias-Machado, Alexandre Kiyoshi Hidaka, Igor Nunes-Silva, Carlos Alberto Chagas, Leandro Correa Leal, Antonio Carlos Lima Pompeo

**Affiliations:** 1 Departamento de Urologia, Faculdade de Medicina do ABC - FMABC - Santo André, São Paulo, Brasil; 2 Departamento de Urologia do Meridional Hospital - Cariacica, Espírito Santo, Brasil

## Abstract

**Introduction:**

In special situations such as malrotated or ectopic kidneys and UPJ stenosis treatment of renal lithiasis can be challenging. In these rare cases laparoscopy can be indicated.

**Objective:**

Describe the Laparoscopic-assisted rigid nephroscopy performed via transpyelic approach and report the feasibility.

**Patients and methods:**

We present two cases of caliceal lithiasis. The first is a patient that ESWL and previous percutaneous lithotripsy have failed, with pelvic kidney where laparoscopic dissection of renal pelvis was carried out followed by nephroscopy utilizing the 30 Fr rigid nephroscope to remove the calculus. Ideal angle between the major axis of renal pelvis and the rigid nephroscope to allow success with this technique was 60-90 grades. In the second case, the kidney had a dilated infundibulum.

**Results:**

The operative time was 180 minutes for both procedures. No significant blood loss or perioperative complications occurred. The bladder catheter was removed in the postoperative day 1 and Penrose drain on day 2 when patients were discharged. The convalescence was completed after 3 weeks. Patients were stone free without symptons in one year of follow-up.

**Conclusions:**

Laparoscopic-assisted rigid nephroscopy performed via tranpyelic approach can be done safely with proper patient selection and adherence to standard laparoscopic surgical principles. This approach is an alternative in cases where flexible endoscope is not available and when standard procedure is unlikely to produce a stone-free status.

## ARTICLE INFO

**Available at: www.intbrazjurol.com.br/video-section/tobias-machado_853_854/**

**Int Braz J Urol. 2016; 42 (Video #7): 853-4**

